# What Do We Know So Far About Ventricular Arrhythmias and Sudden Cardiac Death Prediction in the Mitral Valve Prolapse Population? Could Biomarkers Help Us Predict Their Occurrence?

**DOI:** 10.1007/s11886-024-02030-9

**Published:** 2024-03-20

**Authors:** D. Dziadosz, L. Daniłowicz-Szymanowicz, P. Wejner-Mik, M. Budnik, B. Brzezińska, P. Duchnowski, K. Golińska-Grzybała, K. Jaworski, I. Jedliński, M. Kamela, J. Kasprzak, M. Kowalczyk-Domagała, K. Kurnicka, D. Kustrzycka-Kratochwil, K. Mickiewicz, O. Możeńska, Z. Oko-Sarnowska, M. Plewka, A. Polewczyk, B. Uziębło-Życzkowska, K. Wierzbowska-Drabik, R. Wachnicka-Truty, E. Wołoszyn-Horák, P. Szymański, A. Gackowski, K. Mizia-Stec

**Affiliations:** 1 1st Department of Cardiology, Faculty of Medicine, Medical University of Silesia, Katowice, Poland; 2 Centre of European Reference Network of Heart Diseases - ERN GUARD-HEART, 47 Ziołowa St, 40-635 Katowice, Poland; 3 Department of Cardiology and Electrotherapy, Faculty of Medicine, Medical University of Gdańsk, Gdańsk, Poland; 4 1st Department of Cardiology, Medical University of Lodz, Bieganski Hospital, Łódź, Poland; 5 1st Chair and Department of Cardiology, Medical University of Warsaw, Central Clinical Hospital, 1a Banacha St, 02-97 Warsaw, Poland; 6 Department of Cardiology, T. Marciniak Hospital, Wrocław, Poland; 7 Cardinal Wyszynski National Institute of Cardiology, 04-628 Warsaw, Poland; 8 Dept of Coronary Disease and Heart Failure, Noninvasive Cardiovascular Laboratory, Medical College, Jagiellonian University, St. John Paul II Hospital, Cracow, Poland; 9 Department of Coronary Artery Disease and Cardiac Rehabilitation, National Institute of Cardiology, Warsaw, Poland; 10 Medicor, Powstańców Wielkopolskich 4, 61-895 Poznań, Poland; 11 Department of Cardiology, Hospital of the Ministry of Interior and Administration, Rzeszów, Poland; 12 Pediatric Cardiology Department, The Children’s Memorial Health Institute, Warsaw, Poland; 13 Department of Internal Medicine and Cardiology, Medical University of Warsaw, Infant Jesus Clinical Hospital, Lindleya str. 4, 02-005 Warsaw, Poland; 14 Department of Cardiology, Center for Heart Diseases, 4th Military Clinical Hospital, Weigla 5, 50-981 Wrocław, Poland; 15 Department of Cardiology, Medical University of Bialystok, 15-276 Białystok, Poland; 16 JO Medical Center, Quo Vadis 1/U6, 02-495 Warsaw, Poland; 17 Department of Cardiology, Poznań University of Medical Sciences, Wielkopolskie, 60-355 Poznań, Poland; 18 Department of Interventional Cardiology and Cardiac Arrhythmias, Military Medical Academy Memorial Teaching Hospital of the Medical University of Lodz, Łódź, Poland; 19 Department of Physiology, Pathophysiology and Clinical Immunology, Institute of Medical Sciences, Jan Kochanowski University, Żeromskiego 5, 25-369 Kielce, Poland; 20 Department of Cardiac Surgery, Świętokrzyskie Cardiology Center, Grunwaldzka 45, 25-736 Kielce, Poland; 21 Department of Cardiology and Internal Diseases, Military Institute of Medicine - National Research Institute, Warsaw, Poland; 22 Department of Internal Medicine and Clinical Pharmacology, Medical University of Lodz, Łódź, Poland; 23 Department of Cardiology and Internal Diseases, Institute of Maritime and Tropical Medicine, Medical University of Gdańsk, Gdynia, Poland; 24 Second Department of Cardiology. Specialist Hospital in Zabrze, Medical University of Silesia, Curie-Sklodowskiej str. 10, Zabrze, Poland; 25 Center of Clinical Cardiology, Central Clinical Hospital of the Ministry of Interior and Administration, Warsaw, Poland

**Keywords:** Mitral valve prolapse, Ventricular arrhythmia, Mitral annulus disjunction, Arrhythmic mitral valve prolapse

## Abstract

**Purpose of the Review:**

To summarize currently available data on the topic of mitral valve prolapse (MVP) and its correlation to the occurrence of atrial and ventricular arrhythmias. To assess the prognostic value of several diagnostic methods such as transthoracic echocardiography, transesophageal echocardiography, cardiac magnetic resonance, cardiac computed tomography, electrocardiography, and electrophysiology concerning arrhythmic episodes. To explore intra and extracellular biochemistry of the cardiovascular system and its biomarkers as diagnostic tools to predict rhythm disturbances in the MVP population.

**Recent Findings:**

MVP is a common and mainly benign valvular disorder. It affects 2–3% of the general population. MVP is a heterogeneous and highly variable phenomenon with three structural phenotypes: myxomatous degeneration, fibroelastic deficiency, and forme fruste. Exercise intolerance, supraventricular tachycardia, and chest discomfort are the symptoms that are often paired with psychosomatic components. Though MVP is thought to be benign, the association between isolated MVP without mitral regurgitation (MR) or left ventricle dysfunction, with ventricular arrhythmia (VA) and sudden cardiac death (SCD) has been observed. The incidence of SCD in the MVP population is around 0.6% per year, which is 6 times higher than the occurrence of SCD in the general population.

**Summary:**

Often asymptomatic MVP population poses a challenge to screen for VA and prevent SCD. Therefore, it is crucial to carefully assess the risk of VA and SCD in patients with MVP with the use of various tools such as diagnostic imaging and biochemical and genetic screening.

## Introduction

Mitral valve prolapse (MVP) is a prevalent valvular condition defined as mitral valve leaflets displacement of more than 2 mm above mitral annulus in parasternal long axis view (PLAX) observed on transthoracic echocardiography (TTE) [1]. It has been estimated that 2–3% of the general population is affected by MVP, it is also consistent across ethnic groups. Though there is a strong possibility that we overestimate the occurrence of MVP, with the correct value closer to 1.2% of the general population [2, 3].

MVP is a heterogenous and highly variable phenomenon with several structural phenotypes. There are 3 layers that the mitral valve consists of fibrosa (at the side of the ventricle), atrialis (at the side of the atrium), and spongiosa in the middle. Myxomatous degeneration/Barlow’s disease is a result of the thickening of the spongiosa layer due to proteoglycan infiltration with intimal thickening of the fibrosa and atrialis. It is characterized by mucopolysaccharide infiltration, water retention, and thickening of mitral valve leaflets with thick and elongated chordae tendineae, increased level of type III collagen, and fragmentation of elastic fibers. Myxomatous degeneration is often found in patients suffering from heritable connective tissue disorders such as Marfan syndrome, Loeys-Dietz syndrome, Ehlers-Danlos syndrome, osteogenesis imperfecta, pseudoxanthoma elasticum, and aneurysms-osteoarthritis syndrome. On the opposite side of the spectrum, there is fibroelastic deficiency (FED) with abnormally thin and smooth leaflets and chordae, decreased amount of elastin, proteoglycan, and collagen. FED is related to the thinning of all three layers due to impaired production of collagen, elastin, and proteoglycans, but a slight, local thickening of the leaflet can be present [4, 5]. In between them lies Forme Fruste with slightly thickened leaflets and variability in the structure of chordae tendineae.

On the other hand, we can classify MVP using imaging methods as typical when thickening ≥ 5mm of prolapsing mitral valve leaflets is observed, if leaflets are thinner than 5 mm, we classify MVP as atypical [6–9].

There are several clinical symptoms of MVP, such as excessive fatigue, exercise intolerance, heart palpitations, chest discomfort, and disruption of the autonomic system, manifesting as low blood pressure and syncope, which suggests an increased parasympathetic tone. They are often coupled with psychosomatic components of anxiety and panic attacks. Occasionally, supraventricular tachycardia is observed. On physical examination, we can auscultate mid-systolic click and systolic murmur that can be more prominent when a patient is standing or is performing the Valsalva maneuver [1].

It is key to mention that in light of current knowledge, MVP patients’ outcomes are closely related to the severity of coexisting mitral regurgitation. MVP accompanied by severe and clinically significant MR that requires surgical intervention to restore life expectancy is rare. Therefore, MVP is deemed a mostly benign condition with a high survival rate [10]. However, in recent years, the association between isolated MVP without MR or left ventricle dysfunction, with sudden cardiac death (SCD) has been brought to our attention. Recently published metanalysis by Nalliah et al. shows that in the subgroup of SCD with an undetermined cause of death, 11.7% had MVP detected. The incidence of SCD in MVP population is around 0.6% per year which is 6 times higher than the occurrence of SCD in the general population, estimated to be close to 0.1%. Moreover, around 5% of all SCD cases are of valvular origin [11]. Often asymptomatic MVP population poses a challenge to screening for ventricular arrythmia (VA) and preventing SCD. Implantable cardioverter-defibrillator (ICD) is the recommended form of primary and secondary prevention of SCD in different clinical scenarios. However, current guidelines do not recommend ICD implantation in asymptomatic MVP patients with preserved ejection fraction, which constitutes the majority of MVP population [11, 12]. Sadly, less than 10% of people who experience sudden cardiac arrest will be successfully resuscitated [13].

For those reasons, it is crucial to carefully assess the risk of VA and SCD in patients with MVP with the use of various tools such as diagnostic imaging, biochemical, and genetic screening followed by diligent observation of the patient’s symptoms and active research into possible red flags in their present and past medical history.

## What Do We Currently Know About VA and SCD Prediction in the MVP Population?

The identification of high-risk MVP patients is based on a multidisciplinary approach. Standard ECG, diagnostic Holter, TTE, transoesophageal echocardiography (TEE), cardiac magnetic resonance (CMR), and computed tomography (CT), are used to search for features prognostic of VA and SCD. Even though a wide range of diagnostic tests is available, the assessment of SCD in this diversified population remains challenging. In the juvenile population, the morphology of the mitral valve and sex play a key role. Among the youth, the myxomatous disease was recorded in up to 7% of SCD cases and even in up to 13% in the female subgroup. When the adult population was analyzed, flail leaflets and severe MR or heart failure (HF) were related to the highest SCD occurrence. Arrhythmia can occur irrespectively of MR, active ischemia, ventricular scarring, primary cardiomyopathy, or channelopathy. At first, MVP patients experience frequent but benign arrhythmias that can become lethal with time. This phenomenon is defined as the arrhythmic mitral valve complex or phenotype (AMVC/AMVP**)** when arrhythmia is frequent (≥ 5% total premature ventricular complex burden-PVC burden) or complex (VT, ventricular tachycardia; VF, ventricular fibrillation) [6••].

### Electrocardiography

When stratifying the risk for arrhythmic events, electrocardiographic monitoring cannot be omitted. Regarding MVP, abnormalities of the repolarization phase are of particular interest. The abnormal stretch of papillary muscles and surrounding myocardium is the reason. T wave inversion (TWI) and QT-prolongation are the hallmarks.

QT-prolongation is related to ventricular arrhythmia, irrespective of MVP, but MVP patients often have longer QT than the control population [14–16]. Furthermore, elongated QT is more frequent if mitral leaflets are thicker and prolapse is severe. It correlates with higher VA risk [17, 18]. Even though QT prolongation is known to increase the SCD risk in the general population, its exact impact on MVP patients’ survival is yet to be determined. TWI is usually present in the inferior (II, III, aVF) and lateral (I, aVL, V5, V6) leads. Over 65% of MVP patients have TWI or bi-phasic T waves. This finding is also associated with malignant arrhythmias among MVP patients [19].

PVCs are signs of significant burden, but non-sustained VT (nsVT) is more related to the SCD risk. PVC morphology exclusive to or characteristic of MVP has not been established yet. One study reported specific morphology originating from the papillary muscles and right ventricular outflow tract in patients with mitral prolapse and successfully resuscitated from cardiac arrest. However, we need to perform more extensive studies to confirm this observation as typical for MVP [20].

Furthermore, fragmented QRS is the notching of the S or R wave, additional *R′*, or at least 2 *R′* in two corresponding leads. Generally, it is thought to be a marker of focal myocardial necrosis. It could be related to complex VAs among MVP patients [21].

**Fig. 1:**
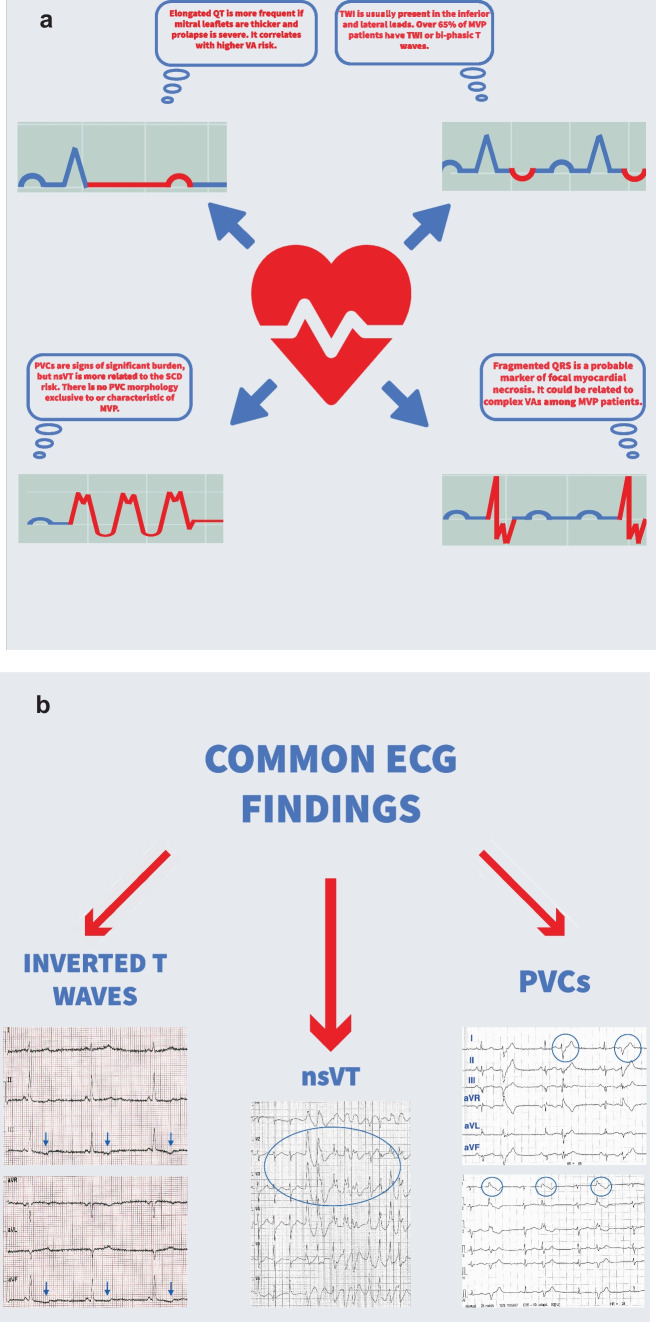
**a** Arrhythmia risk factors found on ECG. **b** Common ECG findings

Continuous Holter ECG monitoring of the electrical activity of the myocardium will provide us with more information than repeated ECG tracings, especially because many MVP patients are asymptomatic. Despite the lack of symptoms, VAs are present in almost 85% of MVP subjects. Although complex VAs are rare, they significantly increase the risk of death. Red flags that one should take notice of are multifocal ventricular ectopy, coupled PVCs, fast nsVT, and presyncope or syncope with frequent or complex VAs without a known cause [22••] (Fig. 1a, b).

### Cardiac Imaging

Several pathomorphological phenotypes of MVP as we have introduced above can be visualized by imaging methods.

Thickened (≥ 5mm) and redundant leaflets, bi-leaflet, or multisegmental prolapse are typical for AMVP. These patients have high arrhythmic risk even when coexisting MR is not severe [17, 23–25].

Bi-leaflet MVP could be related to SCD; however, current cohort studies do not support the statement [26]. Still, this phenomenon causes stretching, which leads to mechanical myocardial stress and fibrosis. It is a component of malignant MVP. It stands for mitral valve prolapse with mild MR and normal left ventricle (LV) dimensions but with significant VA burden of complex VAs and PVCs with right bundle branch block (RBBB) morphology originating from the LV outflow tract, papillary muscles, or fascicles [27]. Basso et al. presented results of anatomical studies in which higher prolapse distance correlated with complex VA incidence [28]. Akcay et al. discovered a relationship between anterior leaflet length and VT [29].

Morphology of the mitral apparatus is crucial for SCD risk assessment. However, the LV function and possible myocardial remodelling must also be evaluated. Especially the arrhythmogenic foci of VA are localized in the left ventricle. It is known that volume overload of the LV caused by chronic, primary mitral regurgitation leads to dilation of the left ventricle and left atrium (LA) to maintain stroke volume. This stage is characterized by normal or even abnormally high LV ejection fraction (LV EF), and LV diameter over the upper limit for the sex and age. Over time compensating mechanisms are not enough, and stroke volume decreases with little impact on the preserved LV EF. However, it has been observed that LV dilation and remodelling can occur even when MR is non-significant or even non-existent. Especially in patients with Barlow’s disease and/or bi-leaflet prolapse [30–32]. Currently there is no clear explanation as to why there is such a disproportion between LV diameter and MR severity. “Prolapse volume” theory could be a possible answer. El-Tallawi et al. proposed that LV burden is the sum of the MR volume and the prolapse volume, which is defined as the end-systolic volume between the mitral annular plane and prolapsing leaflets. Prolapse volume is especially high in myxomatous degeneration (Barlow’s disease) and bi-leaflet prolapse. Other studies reported similar results with the use of CMR and echocardiography [33–35]. Moreover, when LV and LA dilate, they are predisposed to arrhythmia like atrial fibrillation or PVCs. This leads to non-ischemic cardiomyopathy and further predisposes to serious arrhythmic episodes that could lead to cardiac arrest and SCD. Another possible trigger for cardiac remodelling is related to the molecular level. Changes in extracellular matrix composition, level of collagen and metalloproteinases as well as inflammatory cytokine signalling could be the reason and will be discussed at large in further sections of this paper.

Apart from the global remodelling, we must take into consideration local structural changes in the myocardial tissue. Focal hypertrophy, mostly in the basal inferolateral region accompanied by fibrosis is prevalent in MVP population [28, 36]. When LV dilates, the mitral annulus gets dilated as well, which results in higher wall tension, according to Laplace’s Law [37].

Mitral annulus disjunction (MAD) is present in almost half of the MVP cases. It is still unknown if MAD develops over time or is congenital. It is a systolic separation between the myocardium and the mitral annulus, reinforcing the PML [38]. This separation results in the loss of mechanical function of the annulus and electrophysiological separation between the left atrium and the ventricle [17, 39, 40]. MAD can be observed on TTE, TEE, CMR, and CT [41–43]. What accompanies MAD is curling. The abnormal systolic movement of the posterior mitral annulus and hypertrophied postero-basal myocardium. We can observe curling on TTE and as a “ballerina foot” sign on angiography. Perazollo found that curling is related to MAD and leads to hypermobility of the annulus. Moreover, there is a correlation between MAD and curling severity. This phenomenon could lead to increased systolic annulus diameter, myxomatous degeneration, and myocardial stretch resulting in hypertrophy and fibrosis [39]. MAD can be measured by TTE in the PLAX view, two and four-chamber views, and CMR in the sagittal view [42]. When TTE measurements are not viable, we can measure MAD on TEE [44]. Even though the presence of MAD is not detrimental, its presence in symptomatic MVP patients, especially in Marfan and Loeys-Dietz syndrome, is related to an increased risk of arrhythmic events or mitral valve intervention [45, 46]. Additionally, arrhythmic mitral valve phenotype often includes MAD. A meta-analysis published in 2021 further proved the relationship between MAD and arrhythmia. Six studies with 848 individuals have shown that the MVP group with complex VAs had a higher prevalence of MAD on TTE than the MVP group without complex VAs [24]. Other studies showed similar results. When MAD is wider, the incidence of nsVT on Holter ECG is higher [47]. Even in the absence of MVP, MAD occurrence is related to aborted cardiac arrests and VT [48]. In summary, MAD is not only a predictor of VA in the context of MVP but could be an independent marker of high arrhythmic risk.

When hypercontractility of the lateral mitral annulus occurs, we can observe a spike in the tissue Doppler imaging. This spike is called a “Pickelhaube sign.” It is the result of curling of the posterior mitral annulus, redundant leaflets, and elongated chordae. Pickelhaube sign is more common in AMVP. It is thought to be a trigger for PVCs, nsVT, and SVT. Abrupt mechanical traction could be responsible for electrical dysfunction [27, 49].

In most MVP cases global longitudinal strain (GLS) can be preserved or slightly changed. Studies in general have not shown a difference between LV GLS in MVP patients compared to controls. However, slight changes in the value of the pre-stretch index (PST) and post-systolic index (PSI) were observed in patients with MVP [50]. Research of the 543 MVP patients with recorded VA and 67 non-VA MVP patients has shown that the VA group had larger mitral annulus diameter, lower LV GLS, higher PST, PSI, and higher frequency of MAD [51]. However, current guidelines accentuate the need for further research into the effectiveness of GLS in this clinical scenario.

Another promising tool would be myocardial work (MW) and myocardial work index (MWI). It is based on speckle tracking and reflects myocardial energy needs and oxidative stress. It could help us identify systolic dysfunction of the hypertrophied and fibrotic segments of the myocardium observed on CMR.

CMR allows to not only evaluate myocardial function but also myocardial morphology, including MAD. Late gadolinium enhancement (LGE) can localize myocardial fibrosis non-invasively. What we are looking for is a pattern that is patchy or diffused. Which means it is of non-ischemic origin that is more characteristic of MVP. Nowadays, the notion that fibrosis is an arrhythmic substrate is well-known and accepted. In MVP, fibrosis generally occurs close to the mitral annulus at the base of the LV wall, papillary muscles, and inferior wall. Diffused fibrosis observed on T1 mapping is closely related to the complex VA occurrence [52]. Recently, it has been observed that replacement fibrosis was more prevalent in the MVP cohort, especially in the bi-leaflet prolapse group. In this study elevated extracellular volume (ECV) which is a surrogate of diffused interstitial fibrosis related to the MVP was associated with MR severity, but not with MVP on multivariable analysis [53]. In particular, myocardial fibrosis affecting both the infero-basal LV free wall and the papillary muscles has been recognized in pathological and LGE-CMR studies. The scarring process has been related to mechanical stretch secondary to the excessive mobility of the mitral valve apparatus due to mitral valvular disjunction and posterior systolic curling. It indicates a promising role of CMR for arrhythmic risk stratification beyond traditional electrophysiological markers [12].

Another parameter that can be obtained with the use of CMR is the angle measured between mitral annular planes at the end-diastole and end-systole. It indicates increased excursion of the posterior annulus to the mitroaortic junction. This angle is increased in MVP patients, irrespectively of MR [54].

**Fig. 2:**
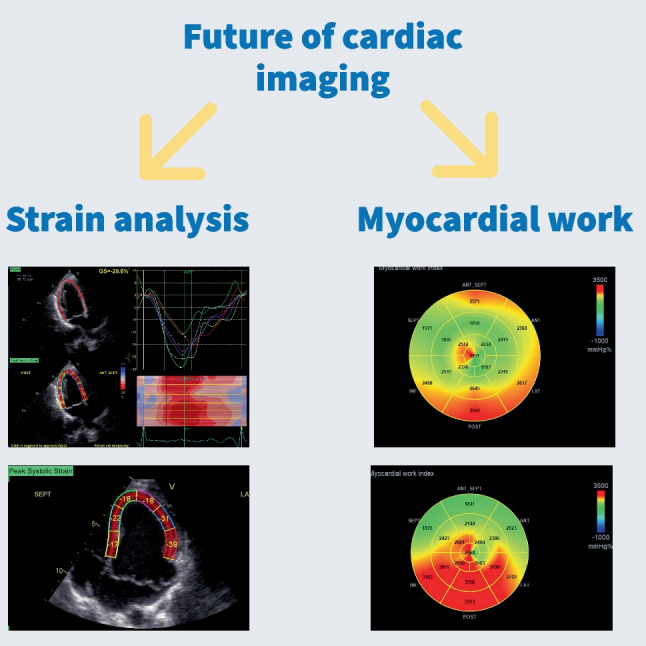
Modern cardiac imaging methods

Unfortunately, CMR and advanced echocardiographic tools such as strain analysis and myocardial work are not readily accessible in daily clinical practice (Fig. 2).

Although MVP often coexists with non-significant MR, it is crucial to monitor its possible progression because severe MR is related to a poor outcome. Degenerative MR with LV dysfunction is related to high SCD risk and increased mortality [26, 29, 39]. The way we quantify MR is by the use of Doppler Echocardiography with LV measurements. TEE is the most sensitive for MR estimation. This way, we can acquire regurgitant volume and effective regurgitant orifice area (EROA) measurements. EROA ≥ 40 mm^2^ and regurgitant volume ≥ 60 mL/beat are characteristics of severe MR. However, mortality increases when EROA reaches 20–30 mm^2^. When LV dysfunction reaches a value of EF < 60% and LV end-systolic diameter ≥ 40 mm, mitral valve surgery should be performed to restore life expectancy [55–57] (Table 1).

**Table 1:**
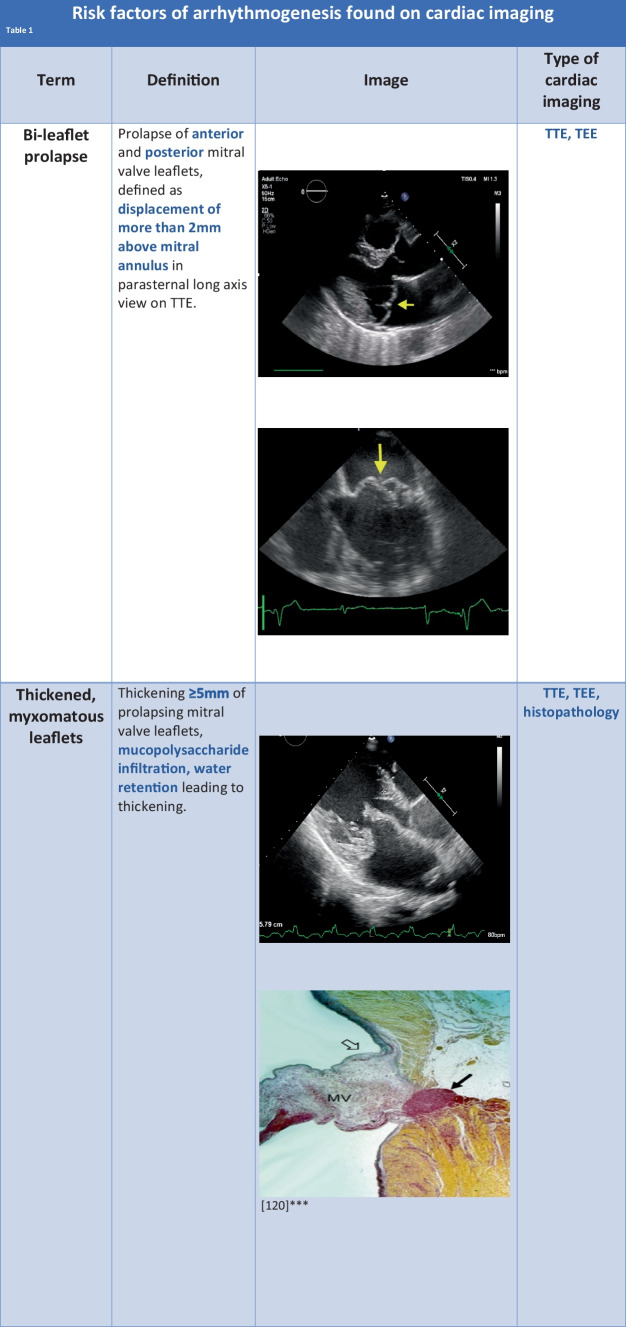

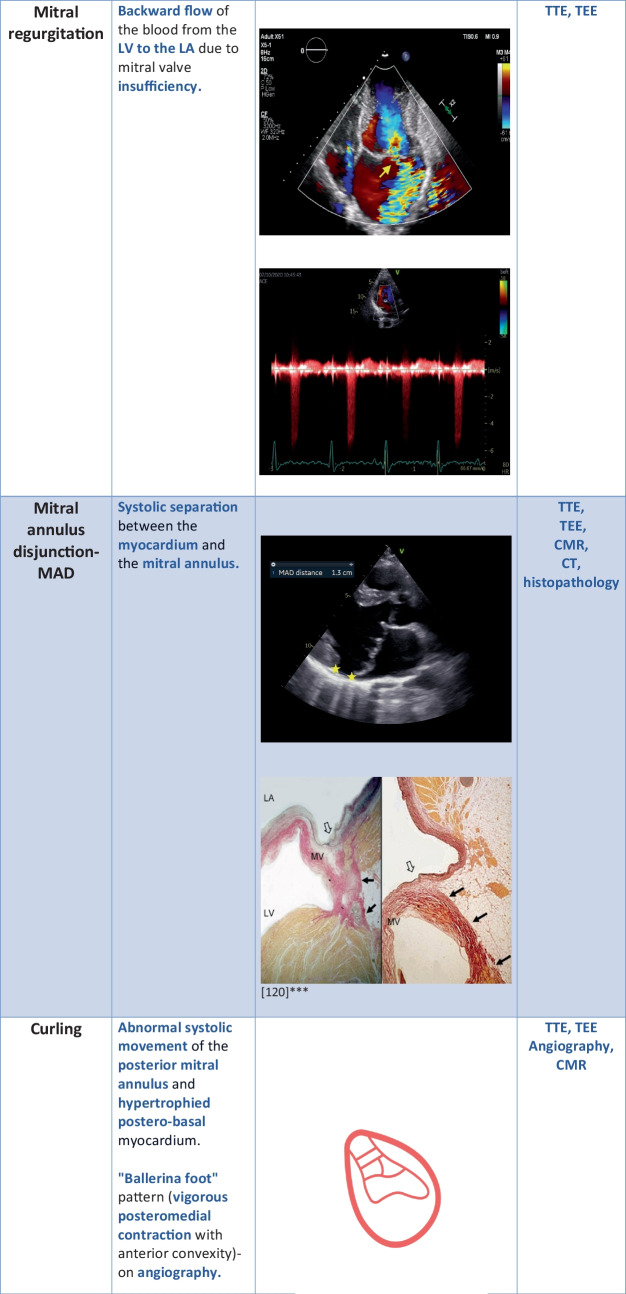

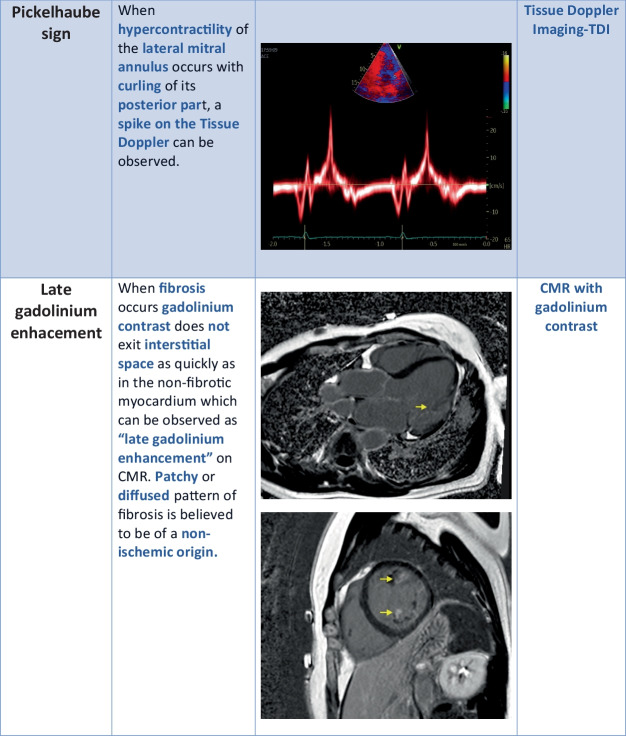
Risk factors of arrhythogenesis found on cardiac imaging

^***^The asterisked images in Table 1 are reproduced from [116], https://www.mdpi.com/2308-3425/8/2/9, Creative Commons user license https://creativecommons.org/licenses/by/4.0/

### Genetic Testing

Another factor that could be a trigger for remodelling is underlying, genetic cardiomyopathy, like previously mentioned connective tissue disorders such as Marfan syndrome, Loeys-Dietz syndrome, Ehlers-Danlos syndrome, osteogenesis imperfecta, pseudoxanthoma elasticum, and aneurysms-osteoarthritis syndrome. A meta-analysis of six genome-wide studies, including 4884 cases and 434,649 controls revealed 14 loci associated with MVP and identified potential MVP-related genes: LMCD1, SPTBN1, LTBP2, TGFB2, NMB, and ALPK3. The authors of the study created a polygenic risk score for this population and presented an improved risk stratification when added to sex, age, and clinical risk factors [58]. It is highly possible that genetic burden with the addition of external factors may result in remodelling, similar to dilated or pregnancy-related cardiomyopathy [59, 60].

### Electrophysiology

The electrophysiological study is an invasive method for SCD risk assessment and could be a therapeutic option if VAs occur and may constitute a valuable tool in the MVP population. In a recent study (Aabel, 2023), arrhythmic mitral valve syndrome patients were monitored for VAs by implantable loop recorders (ILR) and secondary preventive ICDs. During 3.1 years of follow-up, severe VA was recorded in 12% with implanted ILR and in 20% of patients with ICD (re-event incidence rate 8% per person-year, 95% CI 3–21). In the ILR group, severe ventricular arrhythmia was associated with frequent premature ventricular complexes, more non-sustained ventricular tachycardias, greater left ventricular diameter, and greater posterolateral mitral annular disjunction distance. Frequent premature ventricular complexes, non-sustained ventricular tachycardias, greater left ventricular diameter, and greater posterolateral mitral annular disjunction distance were predictors of the first severe arrhythmic event [61].

**Fig. 3:**
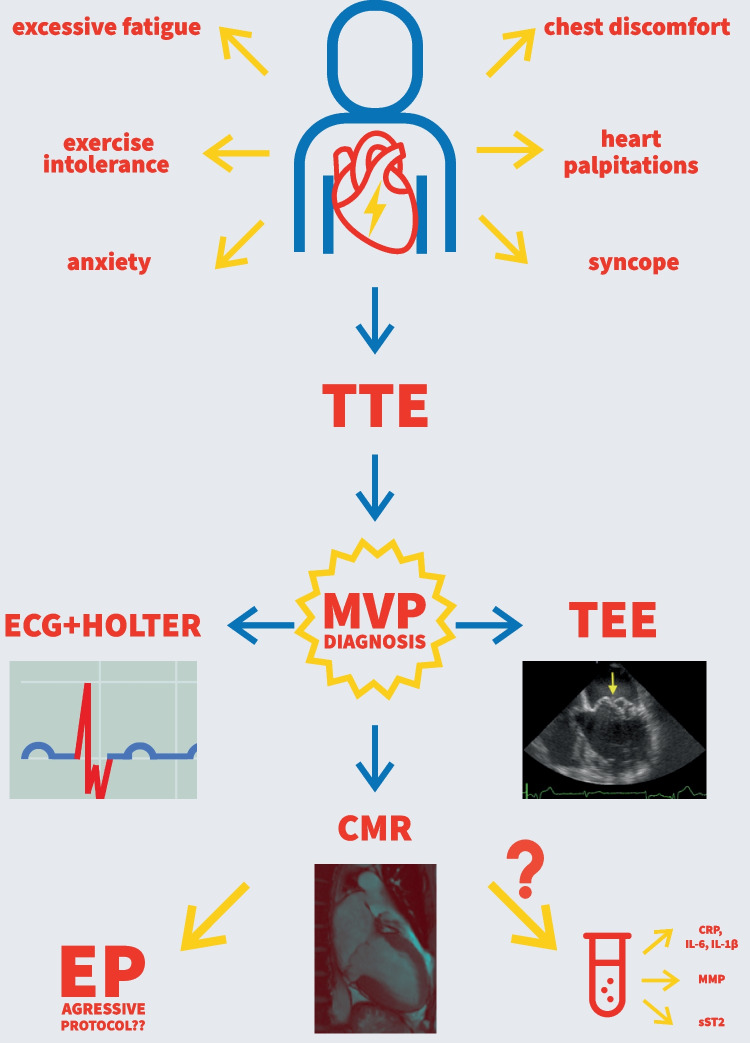
Diagnostic path of an MVP patient

Several studies presented results of EP in patients with MVP and revealed conflicting results. When VA was induced, it mainly originated from papillary muscles (PMs) and Purkinje cells [20, 62]. According to the other authors, VA arose from PMs and inferobasal region and the rest reported a multifocal arrhythmia. These findings correlate with the notion of diffused fibrosis being a hallmark of MVP. In theory, catheter ablation of typical inferobasal regions of the myocardium would allow us to decrease the arrhythmic burden. Though it is highly possible that it would be a short-term solution with new areas being the arrhythmogenic zones. This is a major downside because the patient would possibly require multiple interventions that are not without risk and do not grant a 100% success rate, especially if the arrhythmic substrate is diffused in the myocardium. Currently, in some individuals with MVP, palpitations and a history of syncope an aggressive EP protocol may be considered. In subjects, in whom monomorphic sustained VT (sVT) is triggered by ≤ 3 ventricular impulses or polymorphic VT/VF is triggered by ≤ 2 ventricular impulses ICD implantation may be considered. Until now, there is a lack of strong evidence that would help us identify MVP patients that would benefit the most from EP and catheter ablation [24] (Fig. 3).

## Is It Possible to Predict the Occurrence of VA with the Use of Routine and Novel Biochemical and Molecular Markers?

Several studies have shown that in most cases, SCD in MVP patients is secondary to sustained and complex VAs such as VF or VT [24]. Despite the ongoing effort to screen, predict and prevent VA-induced SCD, there is still room to improve or widen the spectrum of available diagnostic tools. The utility of classic biomarkers such as brain natriuretic peptide (BNP) or troponins is highly prevalent in cardiology. However, their ability to predict VA episodes is unclear, with promising results from published trials [63].

Pathophysiology of VAs has different mechanisms, such as increased automaticity or activity and re-entry. The first two are of cellular origin, and the last is related to the cardiac syncytium. Enhanced automaticity is caused by the acceleration of the firing of action potentials. When the automaticity of myocardial cells is increased, irregular and possibly dangerous patterns can develop, especially in the presence of cardiac disease. The triggered activity of myocardial cells is caused by Calcium (Ca2 +)-mediated premature action potentials that start from early afterdepolarizations (EADs) and delayed afterdepolarizations (DADs). The re-entry phenomenon is the most common cause of VAs. Its origin is multicellular and involves at least two wavefronts propagating around refractory tissue or rotating as spiral waves [64]. Above mentioned mechanisms are related to the overload, neurohormonal activation, inflammation, and necrosis of the myocardium with various signalling proteins. It is possible to measure their level and concentration in the patient’s serum to indicate signalling activation [63].

### Routinely Used Cardiac Biomarkers

When a myocardium suffers from volume expansion and pressure overload, cardiac cells, especially ventricular cells, release natriuretic peptides, i.e., B-type natriuretic peptide (BNP) into the serum. BNP is used to assess the severity and to monitor possible worsening or alleviation of HF. BNP concentration can predict mortality independently of LVEF in patients with coronary artery disease (CAD), chronic HF, asymptomatic LV dysfunction, and acute coronary syndromes.

On the other hand, there is a link between myocardial wall stretch and ion channel activation resulting in arrhythmia. Especially when the wall stretch is rapid and ventricular loading is disturbed due to ventricular dyskinesia or regional muscle traction, as in MVP [65].

There are a lot of studies confirming the role of natriuretic peptides in SCD risk prediction; however, most of them are dedicated to patients with overt or asymptomatic HF or CAD. Meta-analysis of 14 studies on BNP and its efficacy in indicating VA or SCD revealed that during a mean follow-up of 19 months, the occurrence of SCD correlated positively with increased BNP. Moreover, among 1047 patients with ICD, after a follow-up of 12–33 months, 261 patients met the primary endpoint which was more common in patients with increased serum BNP. The primary endpoint was classified as appropriate ICD therapy for VT or VF, the occurrence of VT, SCD, or appropriate ICD shock therapy. In studies included in the meta-analysis that used multivariate models, BNP predicted VA or SCD with a lower *p*-value than LV EF [66]. In a group of 398 patients with HF in NYHA class III and IV and LV EF < 40%, increased BNP was related to QTc interval prolongation. There was no difference in SCD rate between groups regarding BNP levels. However, SCD correlated with QTc prolongation. Kaplan–Meier SCD survival rate was 2.9 times higher when there was no QTc prolongation. BNP with QTc prolongation could be beneficial in evaluating the risk of SCD in severe HF populations [67]. There are other studies that presented the association between BNP and NT-proBNP levels and the high risk of VT occurrence [68].

Although we have no data on natriuretic peptides and the risk of SCD in MVP group, there are some studies in asymptomatic populations. The Nurses’ Health Study was a prospective cohort investigation of 121,700 female nurses aged 30–55 at baseline. Sudden arrhythmic death was a study endpoint, follow-up lasted 16 years. The mean time between study enrolment and SCD was 120.5 months. During the observation period, 99 SCD cases were recorded and matched to 294 controls adjusted for CAD, glomerular filtration rate, and other biomarkers. Out of 99 women, 30 had CVD. The baseline NT-proBNP value was associated with SCD occurrence. The correlation was independent of other comorbidities and risk factors of CVD. Thus, NT-proBNP used with other cardiac risk factors could help us predict SCD in long-term observation of the female population [69].

Cardiac troponins (cTns): C, I, and T (cTnC, cTnI, cTnT) in the situation of overload or injury are released into the bloodstream. All three of them are markers of myocardial damage. The association between increased cTn levels and mortality in HF and CAD patients is well-known [70, 71]. The predictive value of cTn concentration among the CVD-free and asymptomatic general population is of particular interest. Dallas Heart Study of a multiethnic general population of 3546 individuals aged 30–65 years, with a 7-year follow-up, determined that increased cTnT was associated with structural heart disease and subsequent risk of all-cause mortality [72]. Another community-based study of 1203 men free of CVD at baseline reported that high cTnI predicted death and the first CAD event. This study accentuates the importance of cTns measurement to detect silent myocardial damage [73]. Garg et al. reported that longitudinal changes in NT-proBNP and hs-cTnT were associated with arrhythmic episodes in 1930 patients who wore a leadless ECG monitor during ambulatory visits. When adjusted for demographics, body mass index, smoking, diabetes, hypertension, stroke, left ventricular mass, cardiac medications, and patch wear time, each increase in NT-proBNP and hs-cTnT was associated with increased risk of ns-VT, atrial tachycardia, and PVC burden [74].

A nested case–control study of 6 cohort studies with 1655 subjects, 565 SCD cases, and 1090 controls reported that several common biomarkers like total cholesterol to high-density lipoprotein cholesterol ratio, cardiac troponin I, NT-proBNP, and hs-CRP were independent and cumulative risk factors of SCD [75]. This study is important to mention because the concentration of the biomarkers correlated with SCD occurrence even in CVD-free subjects. These findings are especially relevant for the MVP population, which often has mild or no symptoms and normal LVEF.

The above-mentioned markers are widely available for assessment in daily clinical practice; however, their cut-off points of normal values or values determining the SCD risk assessment were established for non-MVP populations. The potential common mechanism of LV overload may argue for further studies on this topic (Table 2).

**Table 2:** Routinely used biomarkers that could help predict the risk of arrhythmia and sudden cardiac death

**Biomarker**	**Association with VA and SCD**	**Was the efficacy of the biomarkers’ concentration evaluated in the MVP population?**	**Performed studies**
**C-reactive protein (CRP), IL-6, IL-1β**	o Neutrophil count, neutrophil/lymphocyte ratio (NLR), platelet/neutrophil ratio (PLR), and monocyte/high-density lipoprotein ratio (MHR) were significantly higher in the MVP population o Lymphocyte/monocyte ratio (LMR) was significantly lower in the MVP group o High basal levels of CRP are related to increased risk of SCD o Increased serum concentration of CRP was related to malignant arrythmia occurrence o Even a slight increase in CRP is related to a 2.8-fold increase in SCD risk o hsCRP > 3 mg/L is related to significantly higher amount of appropriate ICD interventions o State of local inflammation leads to prolonged action potential duration and repolarization in the affected area o In the state of global inflammation QT prolongation can be observed	**Yes**	*Evaluation of inflammation markers in mitral valve prolapse* *Prospective study of C-reactive protein, homocysteine, and plasma lipid levels as predictors of sudden cardiac death* *Injury and Inflammation Markers Evaluation to Predict Clinical Outcomes After Implantable Cardioverter Defibrillator Therapy in Heart Failure Patients with Metabolic Syndrome* *Prospective study of C-reactive protein, homocysteine, and plasma lipid levels as predictors of sudden cardiac death* *Mechanistic Insights Into Inflammation-Induced Arrhythmias: A Simulation Study*
**B-type natriuretic peptides: BNP, NT-proBNP**	o When a myocardium suffers from volume expansion and pressure overload, cardiac cells release natriuretic peptides into the serum o BNP can predict VA or SCD with a lower p-value than LV EF o Increased BNP is related to QTc interval prolongation which correlates with SCD occurence	**No**	*Brain natriuretic peptide for the prediction of sudden cardiac death and ventricular arrhythmias: a meta-analysis* *Relation of B-type natriuretic peptide level in heart failure to sudden cardiac death in patients with and without QT interval prolongation* *B-type natriuretic peptide is a major predictor of ventricular tachyarrhythmias* Amino-terminal pro-B-type natriuretic peptide and high-sensitivity C-reactive protein as predictors of sudden cardiac death among women
**Cardiac troponins: cTnC, cTnI, cTnT**	o cTns are released into the bloodstream when injury or stress occurs o High serum concentration of cTnT is associated with structural heart disease and subsequent risk of all-cause mortality o Increase in NT-proBNP and hs-cTnT is associated with an increased risk of ns-VT, atrial tachycardia, and PVC burden	**No**	*Troponin I as a predictor of coronary heart disease and mortality in 70-year-old men: a community-based cohort study* Association of Longitudinal Changes in Cardiac Biomarkers With Atrial and Ventricular Arrhythmias (from the Atherosclerosis Risk in Communities [ARIC] Study)

### Markers of Inflammation

In the MVP population, subclinical local inflammation could be a result of LV damage. On the other hand, every generalized inflammation of different origin has pro-arrhythmogenic properties and an increased concentration of inflammatory markers should be considered a red flag for arrhythmia occurrence. It is important to treat and prevent any infection, because it may lead to higher arrhythmic risk, regardless of the MVP presence [76].

C-reactive protein (CRP)-classified as acute phase reactant protein, mainly synthesized in the human liver, following the increase of interleukin 6 (IL-6) serum level during an ongoing inflammation or injury. It can be synthesized and released by fatty tissue, smooth muscle, and vascular endothelial cells. The state of the inflammation encourages the “negative cardiac remodelling” that can be a source of arrhythmia. The correlation between the increase of CRP and IL-6 and the formation of unstable atherosclerotic plaques and the correlation between coronary artery disease and SCD has been established [77].

There are no data between CRP and SCD in MVP, thus below we present data on the concentration of CRP, IL-6, and non-CAD related VAs and SCD.

Studies showed the association between increased serum concentrations of inflammatory markers with SCD occurrence. Hussein et al. analyzed 5888 elderly patients, including 3035 patients with no cardiovascular disease (CVD). There were no significant differences regarding the occurrence of SCD in the subpopulation without known CVD regarding CRP level, but differences in serum concentration of IL-6 were significant [78]. Another published study focused on a 22,071 male physician population with no CVD at the time of enrolment. Results of the 17-year follow-up period reported that high basal levels of CRP were related to an increased risk of SCD. Men that measured in the highest quartile regarding CRP concentration had a threefold increase in SCD risk than men in the lowest quartile [79]. On the other hand, a similar study with the female population did not show a significant difference [69]. Moreover, we can analyze data regarding patients with implantable cardiac devices. Results of those analyses have shown that increased serum concentration of CRP was related to malignant arrhythmia occurrence. In those studies, patients with a recent history of cardiovascular events or unstable CAD or heart failure (HF) were excluded from the studied group [80, 81]. There is also a possible correlation between QT-interval prolongation leading to another malignant arrhythmia, Torsades de pointes (Tdp), and increased CRP concentration [82]. We can currently detect low serum concentrations of CRP (< 0.04 mg/L) with high-sensitivity CRP assays (hsCRP) during low-grade inflammation. The previously mentioned epidemiological study showed that even a slight increase in CRP is related to a 2.8-fold increase in SCD risk [79]. Additionally, the population with implanted ICD (primary and secondary prevention) with hsCRP > 3 mg/L had a significantly higher amount of appropriate device interventions in comparison to those with lower levels of hsCRP. It is even postulated that hsCRP level may be an indicator of SCD, more so in the low cardiovascular-risk population than in high-risk patients [83].

The influence of inflammatory cytokines on ion channels located on the surface of cardiomyocytes is called inflammatory cardiac channelopathy. They mainly affect sodium, potassium, and calcium channels without any structural changes in the heart cells but with direct arrhythmic effects. For example, IL-6, IL-1β, and tumor necrosis factor (TNF) have the ability to prolong the action potential duration (APD) and QT interval, which favors the occurrence of VA. Another promising study on a multiscale virtual heart model investigated the impact of pro-inflammatory cytokines on ventricular electrophysiology. In a human cell model, IL-6, IL-1β, and TNF increased the APD. Moreover, the difference between APDs (ΔAPD) in epicardial and middle/endocardial cells was significant in comparison to the control, which leads us to believe that state of inflammation causes higher transmural repolarization heterogeneity. State of local inflammation led to prolonged APD and repolarization in the affected area. Pathological heterogeneity and regional differences in repolarization were substrates to unidirectional conduction blocks, and the risk of re-entry arrhythmia increased. In the state of global inflammation, QT prolongation was observed. The most compelling finding was the impact of globally prolonged repolarization on the decreased adaptability of the myocardial tissue to higher frequencies. It resulted in a 2:1 conduction block, even at physiologically relevant heart rates. It is crucial to mention that APD prolongation can have an antiarrhythmic effect. However, to achieve the therapeutic goal, APD prolongation must be homogenous. What is potentially dangerous is the transmural heterogeneity of APD in the myocardium [84]. As of now, there is only one study that investigated the concentration of markers of inflammation exclusively in the MVP population (461 patients) in comparison to the non-MVP control (459 patients). The results have shown that neutrophil count, neutrophil/lymphocyte ratio (NLR), platelet/neutrophil ratio (PLR), and monocyte/high-density lipoprotein ratio (MHR) were significantly higher in the MVP group and lymphocyte/monocyte ratio (LMR) was significantly lower in the MVP group in comparison to the control. In logistic regression analysis PLR, LMR, and NLR were independent predictors of MVP presence. No information regarding clinical symptoms and arrhythmic episodes was provided in this study [60].

Further investigation into the possible involvement of inflammation markers in the process of arrhythmogenesis will allow us to look for novel and targeted therapies for the treatment and prevention of VAs. The use of monoclonal antibodies or short decoy peptides that divert autoantibodies from their binding sites on ion channels is a promising research route of the future even in subjects with MVP [85] (Table 2).

## Experimental Cardiac Biomarkers

sST2 is one of the new and promising biomarkers that could help us assess the condition of the cardiovascular system. The abbreviation stands for a soluble receptor of Suppression of Tumorgenicity 2 (ST2), a member of the IL-1 receptor family. ST2 has two isoforms, previously mentioned sST2 and ST2L, a transmembrane receptor. IL-33 is a natural ligand of ST2 that acts both as a cytokine and transcription factor. IL-33 gets expressed by smooth muscle and endothelial cells, but most importantly by fibroblasts [86]. ST2L-IL-33 pathway is activated when cardiomyocytes are damaged or during mechanical stress of myocardial tissue. This signalling pathway alleviates hypertrophy, apoptosis, and fibrosis of the myocardium. Although ST2L isoform plays a positive role in the human body, the sST2 form has the opposite effect. During myocardial cell injury, there is an increase in the sST2 serum level. The soluble form conquers with ST2L pathway and negates its cardioprotective qualities [87]. Results of a study published by Scheirlynck et al. have shown for the first time a possible relationship between increased serum concentration of sST2 and the occurrence of VAs in a population with MAD. Patients who suffered from toxic, ischemic, or valvular heart disease (other than mitral valve disease), ACA, or underwent procedures on a mitral valve or had radiofrequency ablation were excluded from the study. Out of 72 patients included in the analysis, 31% had ventricular arrhythmia in the form of aborted cardiac arrest, sustained or ns-VT recorded. Soluble ST2 serum concentration was significantly higher in the VA than in the non-VA subgroup. Moreover, the group that suffered from arrhythmia had a higher prevalence of LGE of papillary muscles. The non-arrhythmic group was characterized by slightly higher LV EF (63% vs. 60%), though in both groups, LV EF oscillated in the normal range. The risk model involving the presence of LGE in the papillary muscles, sST2 serum level, and LV EF was proposed. The addition of these elements had a higher specificity and sensitivity in predicting VA or aborted cardiac arrest (ACA) than one parameter alone. It is worth mentioning that the study reported a possible relationship between CRP and sST2 concentration. CRP level was notably higher when the concentration of sST2 was increased. This is the first time when a new biomarker was used to stratify the risk of VA occurrence in the MVP population [88]. Another interesting multicentre study focused on identifying patients with a high risk of ICD interventions with the use of sST2. The investigated population consisted of 206 patients with implanted ICDs in primary prevention. Almost half of the population had moderate/severe mitral insufficiency, but 60% suffered from HF of ischemic origin. After a one-year follow-up period, the level of sST2 serum concentration at the time of admission to the study was predictive of ICD appropriate therapy (intervention when VA is detected), ICD inappropriate therapy (intervention when supraventricular tachycardia is detected), and overall survival after ICD intervention. The higher the sST2 level, the higher the risk of ICD therapy occurrence, but the survival rate after ICD therapy decreases. The lowest recorded sST2 level correlated with a four times greater survival rate after ICD intervention [87, 80]. MUSIC registry positively reported that higher sST2 levels were associated with SCD in HF patients. Among the subgroup with sST2 concentration > 0.15 ng/mL, 34% suffered SCD. SCD occurred in 74% of the population with increased sST2 with a high level of NT-proBNP [89]. After encouraging results same outcome could not be reproduced, HF-ACTION trial reported a strong correlation between increased sST2 with worsening HF, but the correlation between sST2 and prediction of SCD was weak [90]. MADIT-CRT trial has shown that baseline level of sST2 could not directly predict VA occurrence, but a 10% increase in sST2 serum concentration over 1 year was related to a higher risk of VA [91].

Even though there is conflicting data regarding sST2 efficacy to predict the onset of VA or SCD occurrence, measurement of sST2 and its addition to other “clinical red flags” may be a promising direction for upcoming studies.

Metalloproteinases (MMPs) are proteins mainly involved in ECM turnover but can also be found intracellularly. *As to our knowledge 24 different MMPs have been identified so far.* Based on their structure and function, they were divided into six subgroups. When activated from their primary inactive forms (zymogens), MMPs are involved in various processes, including post-MI myocardial remodelling and regulation of the inflammatory and fibrotic components during myocardial wound healing. Metalloproteinases interact with growth factors and their receptors and participate in cell adhesion and the proteolytic process of cytokines. The activity of MMPs is inhibited and regulated by tissue inhibitors of metalloproteinases (TIMPs). MMPs are currently used to diagnose and treat many cancerous and autoimmune diseases. MMP-1 and TIMP-1/2 can be found in all human heart valves though MMP-2 can only be found in the aortic and pulmonary valves. Currently, the role of MMPs in the CVD pathogenesis is under investigation. Especially regarding HF, cardiomyopathies, and valvular insufficiencies. The relationship between MMPs and MR was explored by several researchers. It was reported by Marc Irqsusi and associates, that in the population with degrees I-III of MR, the degree of MMP expression was correlated with the severity of mitral insufficiency. The studied group comprised 80 patients (33 females and 47 men), aged between 32 and 89 years, with a mean age of 67 at the time of admission to the study. Patients underwent cardiopulmonary bypass surgery for mitral valve reconstruction, followed by obtaining a tissue sample from posterior mitral valve leaflets (PMLs). Microscopic examination revealed that 44% suffered MR as a complication of endocarditis, and 56% had degenerative MR. In the degenerative MR group, 27% had myxoid, 18% had sclerotic, and 11% had mixed (myxoid and sclerotic) degeneration. Expression of MMP-1 was observed in all examined PMLs, and its signal was scored from 1 to 3, MMP-9 was expressed in 94% of PMLs and scored from 0 to 3. TIMP-1 was detected in 86% of the mitral valves and scored from 0 to 3, TIMP-2 was present in 95% of examined tissue and scored similarly, from 0 to 3. Weak signal intensity was classified as a signal expression of 0–1, and 2–3 scored as high signal intensity. There was a strong correlation in the simultaneous expression of MMP-1 and 9 in 84% of samples. A similar correlation was reported between TIMP-1 and MMP-1. Among MR grade I patients, MMP-1 was expressed strongly in 25% (signal expression = 2) and 75% (intensity score 3). In the MR grade II group, MMP-1 expression was weaker, with 11% with a score of 1, 52% having an expression score of 2, and only 37% having a score of 3. In MR grade III, signal expression decreased further. Therefore, the more advanced mitral insufficiency, the weaker MMP-1 signalling. Results of MMP-9, TIMP-1, and TIMP-2 expression were similar to MMP-1 and correlated negatively with MR grade. It is key to consider the significantly lower expression of MMP-1, MMP-9, TIMP-1, and TIMP-2 in patients with degenerative MR than in the endocarditis-related MR, especially in high expression signalling score. When the degenerative MR was investigated further, no significant difference between myxoid, sclerotic, and mixed groups was found regarding MMP and TIMP signal intensity. Even though the results of the study are not completely in favor of MMP and TIMPs as markers of mitral valve insufficiency, they present an interesting topic for research, especially since MMP-9 is usually absent in human heart valves (though it is present in myocardial cells) but was present in those with significant insufficiency and correlated with the grade of MR. Unfortunately, arrhythmic events were not investigated and were not included in the primary or secondary endpoints [92•]. Other observational studies reported the association between MMP-9 and the occurrence of VA, but the etiology of the phenomenon is still unknown. Possible answers include fibrosis, intracellular uncoupling, and calcium imbalance. MMP-9 is a zinc-dependent endopeptidase, like other MMPs it is involved in fibrosis and inflammation during cardiac remodelling, it also degrades ECM. It is secreted by leukocytes, fibroblasts, and myofibroblasts. Clinical studies established that MMP-9 concentration is higher when cardiac dysfunction occurs, for example in patients with HF [93–95]. Higher level of MMP-9 was also related to VA [94–98].  Study on the human induced pluripotent stem cell-derived cardiomyocytes (hiPSC-CMs) has shown that inhibition of MMP-9 results in the inhibition of irregular arrhythmia-resembling calcium transients. It also included animal subjects and delivered compelling results. Firstly, the pleiotropic function of MMP-9 was observed. In mouse models, activation of MMP-9 was associated with VA occurrence. Mice naturally deficient in MMP-9 were significantly less vulnerable to arrhythmia and had reduced myocardial fibrosis. Furthermore, in animal cardiomyocytes and hiPSC-CMs, the decreased activity of MMP-9 prevented calcium leakage (irregular calcium transients) from the sarcoplasmic reticulum via protein kinase A (PKA) and ryanodine receptor phosphorylation. Even though the results of the abovementioned studies seem promising, there is a lack of large clinical studies on the efficacy of MMP-9 inhibition and its antiarrhythmic quality [99] (Table 3).

**Table 3:** Experimental biomarkers that could help predict the risk of arrhythmia and sudden cardiac death

**Biomarker**	**Association with VA and SCD**	**Was the efficacy of the biomarkers’ concentration evaluated in the MVP population?**	**Performed studies**
Soluble receptor of Suppression of Tumorgenicity 2: sST2	o There is a link between an increased serum concentration of sST2 and occurrence of VAs such as ns-VT and aborted cardiac arrest o Arrhythmia and high sST2 level relate to a higher prevalence of late gadolinium enhancement of papillary muscles (LGE) on cardiac magnetic resonance (CMR) o Basal level of sST2 serum concentration could be predictive of ICD appropriate therapy	**Yes**	*Soluble ST2: a valuable prognostic marker in heart failure* *Increased levels of sST2 in patients with mitral annulus disjunction and ventricular arrhythmias* *Injury and Inflammation Markers Evaluation to Predict Clinical Outcomes After Implantable Cardioverter Defibrillator Therapy in Heart Failure Patients With Metabolic Syndrome*
Metalloproteinases: MMPs	o MMPs are involved in post-MI myocardial remodelling and regulation of the inflammatory and fibrotic components during myocardial healing o The degree of MMP expression is correlated with the severity of mitral insufficiency o MMP-9 is usually absent in healthy human heart valves but is present when mitral valves are insufficient. It correlates with the grade of MR o There is a possible correlation between MMP-9 levels and VA o Animal cardiomyocytes naturally deficient in MMP-9 are significantly less vulnerable to arrhythmia and had reduced myocardial fibrosis	**Yes**	*Role of matrix metalloproteinases in mitral valve regurgitation: Association between the of MMP-1, MMP-9, TIMP-1, and TIMP-2 expression, degree of mitral valve insufficiency, and pathologic etiology* *Differential expression of tissue inhibitors of metalloproteinases in the failing human heart* *Upregulation of lysyl oxidase and MMPs during cardiac remodelling in human dilated cardiomyopathy* *Differential expression of tissue inhibitors of metalloproteinases in the failing human heart* *Pleiotropic Effects of Myocardial MMP-9 Inhibition to Prevent Ventricular Arrhythmia*
Galectin-3: Gal-3, Osteopontin: OPN	o Gal-3 is a binding lectin that plays a role in myocardial fibrosis and proliferation o Osteopontin is a glycoprotein found in ECM, produced by macrophages and fibroblasts o Increased Gal-3 concentration results in increased fibroblast proliferation and increased collagen synthesis o High Gal-3 concentration is often accompanied by an increase in hs-CRP and NT-proBNP o Studied group in which VAs were recorded had significantly higher serum concentrations of OPN and Gal-3 than the non-VA subgroup o Gal-3 and OPN could be able to independently predict the occurrence of VA and with OPN being more sensitive o Gal-3 and OPN could be able to predict an appropriate ICD intervention o One study reported that Gal-3 was not an independent predictor of arrhythmic events but had significance in predicting all-cause mortality in the population with HF on non-ischemic etiology	**No**	*Galectin-3: an open-ended story* *Osteopontin and galectin-3 predict the risk of ventricular tachycardia and fibrillation in heart failure patients with implantable defibrillators* *Predictors of Total Mortality and Serious Arrhythmic Events in Non-Ischemic Heart Failure Patients: The Role of Galectin-3* Clinical Impact of Circulating Galectin-3 on Ventricular *Arrhythmias and Heart Failure Hospitalization Independent of Prior Ventricular Arrhythmic Events in Patients with Implantable Cardioverter-defibrillators*
Heart fatty acid-binding protein: H-FABP	o H-FABP plays a role in lipid transport and cardiac metabolism, but its specific role in cardiac remodelling remains unknown o It is released to the bloodstream when myocardial cells get damaged and strained o High H-FABP concentration could be a predictor of ICD shock or intervention like anti-tachycardia pacing	**No**	

## Other Biomarkers

Apart from routinely used biomarkers or MMPs and sST2, there are other promising markers that could be used as VA and SCD risk predictors. However, they were not studied in relation to the MVP population. Therefore, detailed information regarding their efficacy in this application is beyond the scope of this paper.

Galectin-3 (Gal-3) is a β-galactosidase binding lectin that plays a role in myocardial fibrosis and proliferation. Gal-3 is synthesized by activated macrophages, mast cells, and eosinophils in the lung, heart, kidneys, and adipose tissue. It is activated during tissue damage and has both intra and extracellular activity. Intercellularly, it manifests its anti-apoptotic qualities but, in the extracellular matrix (ECM) it is involved in the inflammation process [100]. Gal-3 mediated collagen synthesis results in heightened secretion of collagen I with almost no effect on collagen type III production. Type III collagen fibers have an elastic structure that can recoil and store kinetic energy. Whereas collagen type I is stiff and rigid. When an imbalance between type I and III collagen production occurs, it leads to the myocardium with systolic and diastolic dysfunction. Studies investigating the role of Gal-3 in human hearts also revealed that in hypertrophied myocardium, Gal-3 concentration was significantly higher than in non-hypertrophied hearts [101]. PREVEND study reported higher baseline serum concentration of Gal-3 in females, especially those burdened by CVD, like hypertension (HTN), dyslipidemia, or renal dysfunction. High Gal-3 concentration was also accompanied by an increase in hs-CRP and NT-proBNP. In the population of 7968 patients, Cox regression corrected for age, gender, HTN, diabetes, and hypercholesterolemia has shown that one standard deviation increase of Gal-3 concentration was associated with a 9% increase in all-cause mortality [102].

Osteopontin is a glycoprotein found in ECM, similar to Gal-3. It is produced by macrophages and fibroblasts. OPN is also vital for the differentiation of fibroblasts which are primary mediators of fibrosis and are known as one of the key substrates of re-entry arrhythmia. Several published studies confirmed that Gal-3 could predict the occurrence of life-threatening arrhythmia. A study published in 2022 reported higher Gal-3 concentration in patients with appropriate ICD intervention (AIT) compared to those whose implanted devices neither treated nor detected VT/VF. The group consisted of 91 consecutive patients with implanted ICD in primary prevention at least six months before baseline Gal-3 measurement. Patients with a history of recent (< 6 months) ICD implantation, MI, catheter ablation for VA, or did not receive optimal medical treatment were excluded. AIT was a primary endpoint, defined as ATP or shock when VT/VF was detected. Unplanned hospitalization caused by decompensated HF was deemed a secondary endpoint. Out of 91 patients, 78% were males, 60% had cardiomyopathy of ischemic etiology, and 47% had atrial fibrillation (AF). Again, there was no information regarding valvular defects. Increased baseline Gal-3 concentration was reported in the subgroup in which non-sustained VTs (nsVT) were previously recorded. The difference was statistically significant. The difference in Gal-3 level among VT/VF positive and non-VT/VF subgroups was close to statistical significance. During the follow-up period of 472 ± 107 days, AIT was present in 20% of patients, and in this group, 94% had ATP, 33% had shock therapy, and 29% experienced both. At the time of enrolment in the study, the group that experienced AIT had higher Gal-3 concentration than the group that did not experience AIT during the follow-up. Interestingly NT-proBNP concentration has not shown any statistical significance regarding VA prediction, but an increase in NT-proBNP positively correlated with increased Gal-3 concentration. The ROC analysis has shown that a Gal-3 serum concentration of 13.13 ng/mL can predict the incidence of AIT with 68% specificity and 83% sensitivity. Importantly, Kaplan–Meier analysis demonstrated that Gal-3 ≥ 13.13 ng/mL was able to predict AIT in patients with both ischemic cardiomyopathy (ICM) and non-ischemic cardiomyopathy (NICM) [103]. It is key to cite the research results performed by Kochi et al., who investigated solely the NICM population. The prospective cohort study included 148 patients who underwent extensive clinical and laboratory assessment, including measurement of Gal-3 serum concentration. The primary outcome was defined as follows: the occurrence of arrhythmic syncope, appropriate ICD therapy, sustained VT, or SCD. All-cause death was a secondary outcome. During the follow-up of 941 days, primary endpoints occurred in over 17% of patients and secondary outcomes in 20% of the population. Gal-3 was not an independent predictor of arrhythmic events but had significance in predicting all-cause mortality in the population with HF on non-ischemic etiology [104]. Other studies have not confirmed the efficacy of Gal-3 as a VA risk predictor. When adjusted for BNP, NT-proBNP, or high sensitivity troponin T (hsTnT) and eight profibrotic biomarkers, Gal-3 had no additional predictive value [105, 106].

Despite the results of the above-mentioned studies, Gal-3 measurement is not recommended in any current European Society of Cardiology (ESC) or American Heart Association (AHA)/American College of Cardiology (ACC) Guidelines [105–107, 12, 108].

It was previously recommended in the 2017 ACC/AHA Guidelines for HF with class IIB as an additional risk predictor of VA [109].

Nowadays, we are aware that fibrosis is one of the hallmarks of MVP pathogenesis and substrate for re-entry VAs. Therefore, further prospective trials regarding possible fibrosis markers, including OPN and Gal-3, could help us identify patients with the highest risk of arrhythmic events.

Heart fatty acid-binding protein (H-FABP) is widely present in myocardial cells, it plays a role in lipid transport and cardiac metabolism, but its specific role in cardiac remodelling remains unknown. H-FABP is released to the bloodstream when cardiac cells get damaged or strained due to its small size and unrestricted cytoplasmatic location. Expression of the H-FABP is regulated by the microRNA mir-1 which possibly plays a role in the progression of HF. Studies have shown that H-FABP can be a predictor of cardiovascular events [110–114]. Nonetheless, the amount of research regarding its role in predicting arrhythmic events is scarce. The Japanese study of 107 patients with cardiomyopathy, including 85 patients with non-ischemic cardiomyopathy (patients with Brugada syndrome, CKD, or recent MI were excluded) and implanted ICD has shown that H-FABP may be a promising tool to predict arrhythmic episodes. Again, data regarding valvular diseases was not provided. During the 33-month follow-up, appropriate ICD intervention/shock or SCD was recorded in 31% and 15% of patients. H-FABP greater than 4.3 ng/mL was a significant, independent predictor of mentioned endpoints. Subgroup analysis has revealed that patients with ICD and receiving amiodarone with lower H-FABP serum concentration had a greater combined cardiac event-free time than those with increased H-FABP concentration. Interestingly, the measurement of troponin T concentration had no significance in predicting the negative outcome [115]. H-FABP could be an effective marker of myocardial strain and injury but its ability to predict arrhythmic episodes must be investigated further (Table 3).

## Conclusions

To properly assess the risk of SCD and VA in the general population, we ought to be vigilant and careful while diagnosing and observing patients with MVP. Clinical assessment, electrocardiography, and modern cardiovascular imaging remain fundamental methods in SCD risk analysis in the MVP population. EP and cardiac ablation, ICD implantation, or surgical intervention should be considered in the selected subjects at the end of the diagnostic path.

The above-mentioned data on biochemical markers of SCD in MVP seems promising but it has several limitations. Regarding cardiac biomarkers, the studied groups were highly variable, with several CVDs, and differed significantly from the typical MVP population. Out of 13 mentioned biomarkers only CRP, sST2, and MMPs were investigated in the MVP group or were reported to have an impact on the TTE and CMR findings like MAD or LGE. Whether their monitoring would be helpful in the MVP population remains unanswered and needs evaluation. Similar or common pathophysiological mechanisms of VA argue for further studies.

## Data Availability

Not applicable.
